# Seroprevalence of SARS-CoV-2 IgG antibodies among health care workers prior to vaccine administration in Europe, the USA and East Asia: A systematic review and meta-analysis

**DOI:** 10.1016/j.eclinm.2021.100770

**Published:** 2021-03-08

**Authors:** Ahmed Hossain, Sarker Mohammad Nasrullah, Zarrin Tasnim, Md.Kamrul Hasan, Md.Maruf Hasan

**Affiliations:** a Department of Public Health, North South University, Dhaka, Bangladesh; b Kurmitola General Hospital, Dhaka, Bangladesh; c Global Health Institute, North South University, Dhaka, Bangladesh; d Health Management BD Foundation, Dhaka, Bangladesh

**Keywords:** Covid-19, Healthcare workers, Seroprevalence, SARS-CoV-2, IgG antibodies, IGG, immunoglobulin-g, IGM, immunoglobulin-m, CMI, chemiluminescent microparticle immunoassay, ELISA, Enzyme-Linked Immunosorbent Assay, P, Proportion/Prevalence, CI, Confidence Interval

## Abstract

**Background:**

Knowing the seroprevalence of SARS-CoV-2 IgG antibodies across geographic regions before vaccine administration is one key piece of knowledge to achieve herd immunity. While people of all ages, occupations, and communities are at risk of getting infected with SARS-CoV-2, the health care workers (HCWs) are possibly at the highest risk. Most seroprevalence surveys with HCWs conducted worldwide have been limited to Europe, North America, and East Asia. We aimed to understand how the seroprevalence of SARS-CoV-2 IgG antibodies varied across these geographic regions among HCWs based on the available evidences.

**Methods:**

By searching through PubMed, ScienceDirect, and Google Scholar databases, eligible studies published from January 1, 2020 to January 15, 2021 were included for the systematic review and meta-analysis. The random-effects model was used to estimate the pooled proportion of IgG seropositive HCWs. Publication bias was assessed by funnel plot and confirmed by Egger's test. Heterogeneity was quantified using I^2^ statistics. We performed sensitivity analyses based on sample size, diagnostic method and publication status. The study protocol was registered with PROSPERO (CRD42020219086).

**Findings:**

A total of 53 peer-reviewed articles were selected, including 173,353 HCWs (32.7% male) from the United States, ten European, and three East Asian countries. The overall seropositive prevalence rate of IgG antibodies was 8.6% in these regions (95% CI= 7.2–9.9%). Pooled seroprevalence of IgG antibodies was higher in studies conducted in the USA (12.4%, 95% CI= 7.8–17%) than in Europe (7.7%, 95% CI=6.3–9.2%) and East Asia (4.8%, 95% CI=2.9–6.7%). The subgroup study also estimated that male HCWs had 9.4% (95% CI= 7.2–11.6%) IgG seroconversion, and female HCWs had 7.8% (95% CI=5.9–9.7%). The study exhibits a high prevalence of IgG antibodies among HCWs under 40 years in the USA, conversely, it was high in older HCWs (≥40 years of age) in Europe and East Asia. In the months February-April 2020, the estimated pooled seroprevalence was 5.7% (4.0–7.4%) that increased to 8·2% (6.2–10%) in April-May and further to 9.9% (6.9–12.9%) in the May-September time-period.

**Interpretation:**

In the view of all evidence to date, a significant variation in the prevalence of SARS-CoV-2 antibodies in HCWs is observed in regions of Europe, the United States, and East Asia. The patterns of IgG antibodies by time, age, and gender suggest noticeable regional differences in transmission of the virus. Based on the insights driven from the analysis, priority is required for effective vaccination for older HCWs from Europe and East Asia. A considerable high seroprevalence of IgG among HCWs from the USA suggests a high rate of past infection that indicates the need to take adequate measures to prevent hospital spread. Moreover, the seroprevalence trend was not substantially changed after May 2020, suggesting a slow progression of long-term SARS-CoV-2 immunity. Routine testing of HCWs for SARS-CoV-2 should be considered even after the rollout of vaccination to identify the areas of increased transmission.

**Funding:**

None

**Research in context**

**Evidence before this study**

We searched in PubMed, ScienceDirect, and Google Scholar for peer-reviewed papers and research reports on seroprevalence of anti-severe acute respiratory syndrome coronavirus 2 (SARS-CoV-2) IgG antibodies, using the search words 'seroprevalence', 'anti-SARS-CoV-2 IgG' and 'COVID antibodies' and similar terms up to January 15, 2021. We identified 53 peer-reviewed sero-surveys. In this context, to assess the seroprevalence of anti-SARS-CoV-2 IgG antibodies among health care workers (HCWs), peer-reviewed studies published in high-indexed journals have been considered to reduce heterogeneity.

**Added value of this study**

This research used existing studies to analyze the pooled-prevalence of anti-SARS-CoV-2 IgG antibodies in HCWs employed in Europe, East Asia, and the United States, and the estimates varied across these geographic regions. Moreover, the seroprevalence of IgG was compared across age groups, gender, country-wise infection risk, work-place infection risk, and study period. Our research also uses statistical techniques to estimate the pooled seroprevalence of IgG antibodies in the HCWs while capturing heterogeneity in the estimates. In order to understand the global pattern of natural immunity against this obdurate virus, the study allowed us to visualize the progression of seropositive status of IgG antibodies among HCWs prior to vaccination.

**Implications of all the available evidence**

Our findings highlight that the immunological landscape has not been changed significantly over time, suggesting a slow progression of long-term SARS-CoV-2 immunity. The seroprevalence of SARS-CoV-2 IgG antibodies among HCWs from the USA is higher than in the countries from Europe or East Asia. As the world plans to find a new equilibrium between minimizing the direct impacts of COVID-19 on the infected and indirect impacts on society, such serological study is crucial to providing new insights into disease transmission.

Alt-text: Unlabelled box

## 1 Introduction

The ongoing pandemic of the 2019 novel coronavirus, known as severe acute respiratory syndrome coronavirus 2 (SARS-CoV-2), was first reported in late December 2019 in Wuhan, China [1]. It grew into a full-scale pandemic within weeks and is now continuing its spread across the world, with nearly 80 million reported cases in 190 countries and more than 1.7 million deaths [2]. A few countries have now approved coronavirus vaccines for use, but as people await their roll-out, cases keep rising in many parts of the world [2]. Hence, it is crucial to understand the vaccines' effectiveness in controlling a pandemic.

There are two ways through which a person may become immune to SARS-CoV-2 infection [3]. Catching the disease typically results in natural immunity to the disease for a certain period and another means of being resistant is vaccination. Comparing the above-mentioned groups would be informative to prove vaccine effectiveness, especially for understanding the gap of antibody duration. Many countries have approved and distributed vaccines across the world to control the pandemic [2]. Together with vaccination and natural protection will help to achieve herd immunity, a state where a proportion of a population needs to be immune to an infectious agent [4]. Therefore, it is essential to know the seroprevalence of natural antibodies that might help estimate the time required for a geographical region to achieve herd immunity as well as would partly explain transmission pattern of the disease in a region.

This ongoing pandemic is a significant burden to the health care services and the HCWs, such as doctors, nurses, hospital cleaners, laboratory technicians, etc., as they remain at the highest risk of exposure to the virus [5], [6], [7], [8], [9]. Moreover, HCWs in Europe and the USA are at increased risk of disease exposure as they live in high-risk transmission zone [8], [9], [10]. To plan an adequate public health response for HCWs and anticipate the disease dynamics, the measurement of anti-SARS-CoV-2 antibodies is of utmost importance.

Antibodies are one of our primary defenses against viruses, created to identify particular proteins on the surface of a virus and initiate processes that gradually neutralize and eventually remove them. The serological tests to detect the presence of IgG antibodies may provide a more reliable estimation of the prevalence of SARS-CoV-2 past infection in the population, as is likely to persist for a more extended period after cleaning up the viral infection [11]. The IgG represents the most robust and long-duration antibody against the SARS-CoV-2 virus and can be detected after a median of 14 days (IQR 10–18 days) from the onset of symptoms during infection [11], [12].

Many seroprevalences of SARS-CoV-2 antibodies related studies have now become available [13], [14]. The majority of seroprevalence surveys with HCWs conducted worldwide have been limited to Europe, North America, and East Asia. The prevalence of such antibodies from a large-scale serosurvey conducted over four collection periods in the US ranged from less than 1% to 23% [13]. To date, three meta-analyses of antibody prevalence among HCWs have been published, and the presence of IgG and/or IgM antibodies has been found to vary between 8% and 17% globally [15], [16], [17]. These articles included pre-print articles as well as accounted IgG and/or IgM antibodies. It was not possible to extract the information only on seroprevalence of IgG antibodies in the HCWs from the available meta-analyses which is essential to understand the global trend of persisting antibody rates over time produced by natural penetration. Thus, our objective was to estimate pooled seroprevalence of SARS-CoV-2 IgG antibodies across geographic regions and to investigate the pattern by age-group, gender, infection risk of HCWs, and study period.

## 2 Methods

### 2.1 Search strategy

We searched the PubMed, Google Scholar, and ScienceDirect online databases to select peer-reviewed papers for systematic review and meta-analysis. We screened observational studies (cross-sectional and cohort) to enunciate the seroprevalence of SARS-CoV-2 antibodies among the HCWs. Our search included only articles published from January 1, 2020, to January 15, 2021. The screening language was restricted to English. In Appendix A, a description of search terms is given. We used *Mendeley* citation management software to compile the results of the search. Henceforth, we manually explored references of selected studies to combine all relevant papers to construct the summary estimates. The study inclines with the Preferred Reporting Items for Systematic Review and meta-analysis (PRISMA) guidelines [18]. The protocol was registered in the PROSPERO database (CRD42020219086).

### 2.2 Selection criteria

The principal outcome of the meta-analysis was the pooled-proportion of IgG antibodies in the HCWs. Our systematic review included studies that documented the serum SARS-CoV-2 IgG antibody status among the HCWs as the outcome of interest. The status was obtained as overall, positive, and borderline/negative determined by the respective serological technique, providing a clinical sensitivity and specificity of at least 80%, used in individual studies. Studies using multiple diagnostic tests to define seropositivity, however, not stratifying the HCWs by the methods were excluded. Studies that contained less than fifty HCWs were also not included which might lead to analysis heterogeneity. Research based at hospitals or healthcare centers were further selected for full-text review, excluding community-based studies that might have partly included HCWs. Additional to the above-mentioned criteria, the prospective studies were designated eligible if they were published in journals that were Q1 or Q2 indexed by the SCImago Journal & Country Rank portal (https://www.scimagojr.com). Although for pooled-prevalence estimation we excluded the grey literature (pre-prints, thesis, and dissertation), the pre-prints were followed to perform sensitivity analyses. We considered eliminating articles for more than one rationale. Titles and abstracts of the studies obtained from the database searches were screened independently by three reviewers-ZT, MKH, and SMN. Any discordance was addressed until an agreement was reached or by the arbitration of AH alone.

### 2.3 Data extraction and quality assessment

A pre-specified form was used for data abstraction. To estimate the seroprevalence of IgG antibodies against SARS-CoV-2, principal data were taken on the total number of HCWs quantitatively evaluated for SARS-CoV-2 IgG antibody levels, how many of them were seropositive and negative from the selected published studies as well as the pre-print articles. Additionally, data on the name of the first author, country, study period (start-end month), study design (cross-sectional or cohort), testing method (any method having at least 80% clinical sensitivity or specificity), median or mean age of HCWs (in years), number of female HCWs, number of HCWs at high risk and infectivity risk of HCWs based on work-type (high, intermediate or low) were recorded from the published article only. We considered high-risk HCWs who were reported to have direct patient contact. We further stratified the articles based on country-wise infection level risk (high, moderate or low). A country was defined as high-risk when the infection crossed millions of cases, as moderate-risk when the infection reached between 500,000 cases and a million of cases. The remaining countries were considered as low-risk group. Moreover, HCWs cross-tabulated by age-group and seropositivity status were documented, whenever possible.

The Newcastle-Ottawa Scale (NOS) was used for the assessment of the included studies (Supplementary file 1). The NOS consists of three domains called selection, comparability, and exposure or outcome of interest. Scores reflect the articles' methodological stringency, lucidity, and clarity. We did not, however, exclude any papers based on quality scoring. Besides, the PRISMA statement consists of a 27-item checklist and is given in Supplementary File 2.

Data abstraction and quality assessment from individual studies was primarily executed by three investigators independently (ZT, MKH, and SMN), from October 2020 to January 2021. All the extracted data and respective evaluations were circumspectly verified by AH.

### 2.4 Statistical analysis

We performed data analysis using *meta* and *metafor* packages in the R statistical software (version 3.6.1). We calculated the seroprevalence of IgG antibodies with a 95% confidence interval (CI) for each study. Following, the pooled seroprevalence was estimated using a random-effects model that allows true effect size to vary from study to study. The calculated proportion from each study and the combined effect estimate with 95% CI were represented graphically by a *Forest Plot*. Publication bias was assessed by observing the symmetry of *funnel plots* visually and confirmed by *Egger's test*. Heterogeneity across the selected studies was investigated by *I² statistic*. The I² statistic represents the percentage of total variation across studies due to heterogeneity rather than chance.

Analysis of the subgroups was carried out to determine the pooled prevalence for each group and look for potential explanations of the heterogeneity. Geographical region (Europe, USA and East-Asia), gender (male and female), mean or median age (less than 40 years and 40 years or older), study period (February-April, April-May and May-September), infection risk based on work-place of the HCWs (high and intermediate/low) and country-wise infection risk level (high, moderate and low) were considered for sub-group analysis. Further, the regional differences by gender and age-group were also calculated. We also conducted sensitivity analyses after removing a few studies to evaluate the robustness of the findings based on sample size, diagnostic method and publication status. In addition, we investigated the associated factors for SARS-CoV-2 IgG sero-positive status by gender, age-group, country-wise risk and work-place risk of HCWs. The studies included in this analysis were, however, observational and could not provide evidence of causality.

### 2.5 Role of the funding source

There was no funding source for this study. All authors had full access to all the data in the study and the corresponding author had final responsibility for the decision to submit for publication.

## 3 Results

### 3.1 Identification and selection of studies

**Fig. 1:**
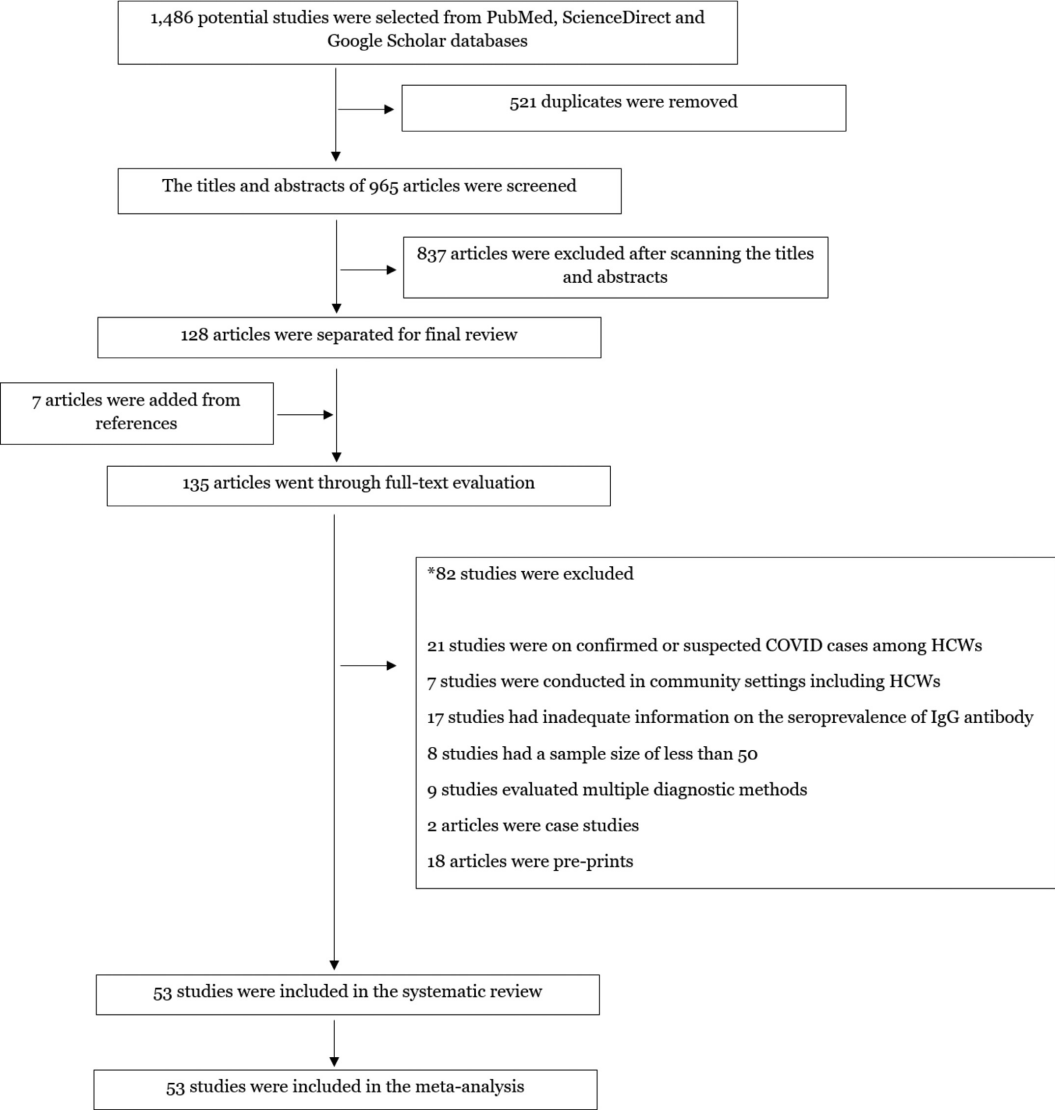
PRISMA flow diagram for study selection.

A flowchart of step-wise literature search to select the appropriate articles is summarized in the PRISMA format and is presented in Fig. 1. The initial search retrieved a total of 1486 studies from the pre-specified databases. After eliminating the duplicates, the titles and abstracts were scanned for further selection of probable articles. Subsequently, the investigators elected 128 articles based on eligibility criteria for full-text review. By manual searching through the included papers’ reference lists, 7 studies were considered for scrutiny, resulting the total number of potential articles to be 135. Finally, 53 studies were included for systematic review and meta-analysis.

### 3.2 Characteristics of the studies

Table 1 outlines the main characteristics of the 53 studies included in our systematic review and meta-analysis. We selected ten countries from Europe, the USA, and three countries from East Asia in the meta-analysis. The majority of the studies (*n* = 34) were conducted in Europe, followed by 12 studies from the USA and 7 studies from East Asia. The East Asian studies were from China, Korea, and Japan. The meta-analysis included 173,353 HCWs, of which 32.7% were male.

**Table 1:** Characteristics of the included studies in the meta-analysis (all the studies were done in 2020).

First author's name listed in alphabetical order	Country	Study Period	Study design	Test method	Total number of health care workers	Median/Mean age (years)	% of Female HCWs	% of high risk HCWs	Infection level
Amendola et al. [33]	Italy	April	Cross-Sectional	ELISA	663	44.0*	83.7	NA	High
Bampoe et al. [34]	UK	May-June	Cross-Sectional	Chemiluminescent Microparticle Immunoassay	200	37.0*	84	100	High
Barallat et al. [35]	Spain	May	Cross Sectional	Chemiluminescent Microparticle Immunoassay	7563	43.8	75.0	NA	High
Black et al. [36]	UK	May-August	Cross Sectional	NA	200	45.3	75	NA	High
Blairon et al. [37]	Belgium	May-June	Cross Sectional	Chemiluminescent Microparticle Immunoassay	1485	NA	73.1	37.03	Moderate
Brant-Zawadski et al. [38]	USA	May-June	Cross-Sectional	NA	2924	42.6	72.7	54.3	High
Brunner et al. [39]	USA	May	Cross Sectional	Lateral Flow Immunoassay	601	NA	72.4	NA	High
Chen et al. [40]	China	January-February	Cross-Sectional	ELISA	105	30.0*	79.04	NA	Low
Corradini et al. [41]	Italy	April	Cross-Sectional	Immunochromatographic Assay	234	43.0*	71.8	NA	High
D. Sims et al. [42]	UK	April-May	Cross Sectional	NA	20,614	43.1	NA	NA	High
Dacosta-Urbieta et al. [43]	Spain	April	Cross Sectional	Immunochromatographic Assay	175	NA	NA	NA	High
Delmas et al. [44]	France	May-June	Cross Sectional	NA	4607	41.8	74.9	NA	High
Duysburgh et al. [45]	Belgium	May-September	Cohort	ELISA	850	NA	NA	NA	Moderate
Fujita et al. [46]	Japan	April	Cross Sectional	ELISA	92	NA	64.1	100	Low
Fernandez et al. [47]	Spain	April-May	Cross Sectional	Chemiluminescent Microparticle Immunoassay	2439	42.1	78.4	NA	High
Godbout et al. [48]	USA	July-October	Cross Sectional	Abbott immunoassay	2217	282	77.3	NA	High
Herzberg et al. [49]	Germany	March-June	Cohort	ELISA	871	39.0	NA	70.1	High
Hibino et al. [50]	Japan	June-July	Cross Sectional	Chemiluminescent Microparticle Immunoassay	806	33.0*	71.6	NA	Low
Hunter et al. [51]	USA	April-May	Cross-Sectional	NA	734	42.8	70.03	NA	High
Iversen et al. [52]	Denmark	April	Cohort	Lateral Flow Immunoassay	28,792	44.4	78.9	4.6	Low
Jeremias et al. [53]	USA	April	Cross Sectional	ELISA	1699	42.8	74.1	NA	High
Khalil et al. [54]	UK	May	Cross Sectional	Chemiluminescent Microparticle Immunoassay	190	NA	NA	NA	High
Ko et al. [55]	Korea	February	Cross Sectional	ELISA	309	31.1	84.5	NA	Low
Kohler et al. [56]	Switzerland	March-April	Cohort	Chemiluminescent Microparticle Immunoassay	1012	38.3*	75.2	20.7	Low
Korth et al. [57]	Germany	March-April	Cross-Sectional	ELISA	316	NA	NA	NA	High
Lackermair et al. [58]	Germany	April	Cross Sectional	ELISA	151	38.0*	83.4	NA	High
Lahner et al. [59]	Italy	March-April	Cross-Sectional	NA	1084	46.0*	NA	55.1	High
Lindahl et al. [60]	Sweden	April	Cross-Sectional	NA	1005	NA	NA	NA	Moderate
Lidstrom et al. [61]	Sweden	May-June	Cross Sectional	Chemiluminescent Microparticle Immunoassay	8679	42.0	76.7	25.6	Moderate
Madsen et al. [62]	USA	April	Cross Sectional	NA	270	NA	NA	NA	High
Mansour et al. [63]	USA	March-April	Cross Sectional	ELISA	285	38.4	45.9	NA	High
Martin et al. [64]	Belgium	April	Cross Sectional	NA	326	NA	NA	82.2	Moderate
Moscola et al. [65]	USA	March-June	Cohort	NA	40,329	42.0*	73.7	45.5	High
Olalla et al. [66]	Spain	April	Cross-Sectional	Immunochromatographic Assay	498	41.5	71.1	NA	High
Pallett et al. [67]	UK	April-June	Cohort	ELISA	6440	41.5	72.0	20.2	High
Piccoli et al. [68]	Switzerland	April	Cross Sectional	NA	4726	41.1	68.3	20.8	Low
Plebani et al. [69]	Italy	February-May	Cross Sectional	Chemiluminescent Microparticle Immunoassay	8285	43.2	71.6	NA	High
Poulikakos et al. [70]	UK	May	Cross-Sectional	Chemiluminescent Microparticle Immunoassay	281	NA	72.9	NA	High
Psichogiou et al. [71]	Greece	April-May	Cross Sectional	Immunochromatographic Assay	1495	46.4	69.7	3.8	Low
Rudberg et al. [72]	Sweden	April-May	Cross-Sectional	Multiplex Assay	2146	44.0	84.6	44.8	Moderate
Schmidt et al. [73]	Germany	April	Cross-Sectional	ELISA	385	NA	80.0	NA	High
Solodky et al. [74]	UK	March-April	Cross Sectional	Lateral Flow Immunoassay	244	NA	NA	NA	High
Sotgiu et al. [75]	Italy	April	Cross-Sectional	Lateral Flow Immunoassay	202	45.0*	65.3	78.2	High
Steensels et al. [76]	Belgium	April	Cross-Sectional	Lateral Flow Immunoassay	3056	NA	NA	35.7	Moderate
Stock et al. [77]	USA	April	Cross Sectional	ELISA	98	37.6	50.0	NA	High
Stubblefield et al. [78]	USA	April	Cross-Sectional	ELISA	249	34.0*	65.5	100	High
Sydney et al. [79]	USA	April-May	Cross Sectional	Chemiluminescent Microparticle Immunoassay	1700	NA	NA	NA	High
Takita et al. [80]	Japan	April-May	Cross Sectional	Immunochromatographic Assay	175	NA	NA	NA	Low
Tu et al. [81]	China	March	Cross-Sectional	ELISA	325	NA	NA	NA	Low
Venugopal et al. [82]	USA	March-May	Cross Sectional	Chemiluminescent Microparticle Immunoassay	478	41.5	68.8	13.6	High
Vlachoyiannopoulos et al. [83]	Greece	April-May	Cross Sectional	ELISA	321	42.7	67.9	NA	Low
Xu et al. [84]	China	March-April	Cross Sectional	NA	4384	NA	73.5	NA	Low
Varona et al. [85]	Spain	April-June	Cross Sectional	Chemiluminescent Microparticle Immunoassay	6038	43.8	71.1	62.7	High

⁎ Median age.

Of the 53 studies, most (90%) of the research designs were cross-sectional, and the other 5 were cohort. Several different test methods have been used to detect the presence of IgG in the blood of health care workers. The enzyme-linked immunosorbent assay (ELISA) and chemiluminescent microparticle immunoassay (CMI) were used most frequently to identify IgG antibody in the included studies. The selected studies were conducted between January and September 2020.

### 3.3 Meta-analysis of the seroprevalence

**Fig. 2:**
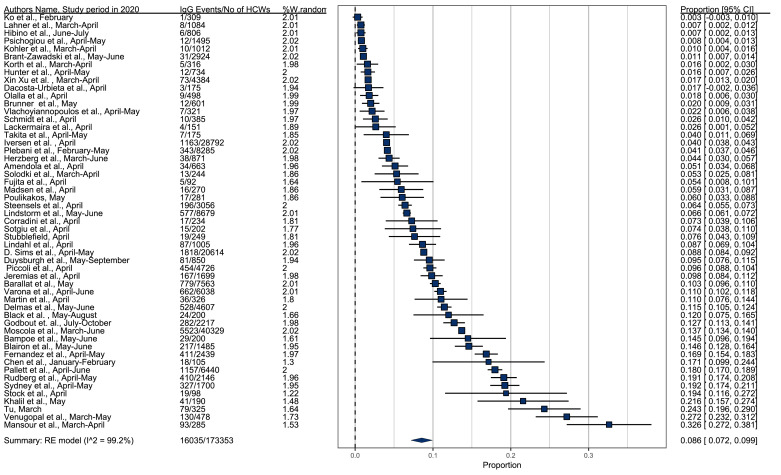
Forest plot of the seroprevalence of SARS-CoV-2 IgG antibodies with corresponding 95% confidence intervals.

The seroprevalence of antibodies against SARS-CoV-2 among HCWs ranged from 0.3% to 32.6% in the studies. Fig. 2 displays the forest plot showing the prevalence of IgG antibody seropositive from studies along with confidence intervals. Estimated by the random-effects model, the pooled serological prevalence of the antibodies was 8.5% (95% CI=7.1–9.9%; I^2^=99.4%). The pooled proportion of IgG seropositivity against the coronavirus was the highest in the USA with 12.4% (95% CI=7.5–17.2%; I^2^=99.7%). However, studies from Europe and East Asia were calculated to have the pooled seroprevalence 7.7% (95% CI=6.3–9.2%; I^2^=99%) and 4.8% (95% CI=2.9- 6.7%; I^2^=95.5%), respectively. There were no precise evidence of publication bias by visual examination of funnel plot symmetry, and further, the absence was supported by the Egger test and is shown in Supplementary file 6.

### 3.4 Subgroup analysis

The subgroup analysis of the seroprevalence by age, gender, study period and infection risk level is given in Table 2. The results indicate an increasing trend in the overall seroprevalence among HCWs over the months from February to September 2020. Globally, the pooled prevalence of antibodies against coronavirus infection in the study period between February and April was 5.7 percent (95% CI = 4.0–7.4%; I2 = 97.7%). During April to May, the prevalence of IgG antibodies increased to 8.2% (95% CI = 6.2–10.0%; I2 = 99.3%) and in the months between May and September, it further increased to 9.9% (95% CI = 6.9–12.9%; I2 = 99.4%) among the HCWs.

**Table 2:** Summary results of subgroup analysis.

Subgroup		No of studies	Pooled seroprevalence	95% CI	I^2^ (%)
Regions	USA	12	0.124	0.078–0.170	99.6
	Europe	34	0.077	0.063–0.092	99.0
	East Asia	07	0.048	0.029–0.067	95.5
Country wise infection level	High	34	0.093	0.073–0.113	99.4
	Moderate	09	0.095	0.065–0.127	98.8
	Low	10	0.039	0.024–0.054	97.9
Gender	Male	25	0.094	0.072–0.116	98.4
	Female	26	0.078	0.059–0.097	99.3
Mean/Median Age	Less than 40 years	12	0.058	0.042–0.074	95.6
	40 years or more	25	0.087	0.068–0.105	99.1
Study period	February-April	10	0.057	0.040–0.074	97.7
	April-May*	28	0.082	0.062–0.100	99.3
	May-September	15	0.099	0.069–0.129	99.4
Work types of HCWs	High risks	18	0.119	0.084–0.154	99.1
	Low or intermediate risks	15	0.086	0.060–0.112	99.6
Overall		53	0.086	0.071–0.099	99.4

⁎ Two of the studies were conducted between March-June.

**Fig. 3:**
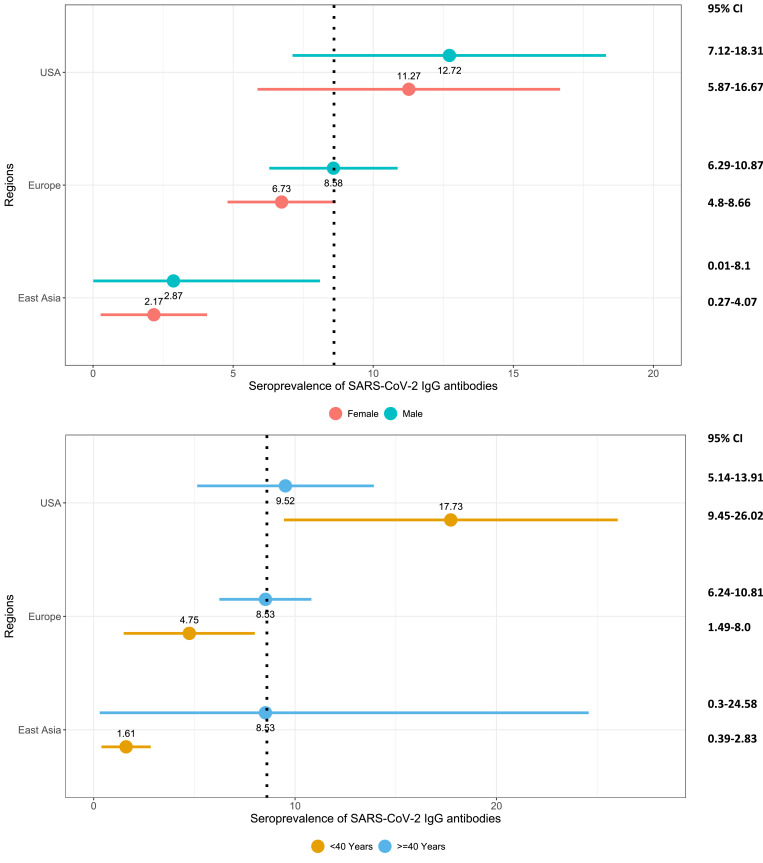
Regional differences of pooled serological prevalence of SARS-CoV-2 IgG antibodies by gender (top) and age group (bottom).

It appears from Table 2 that compared to the female participants (7.8%, 95% CI=5.9–9.7%), the pooled prevalence of IgG antibodies was moderately higher among the male HCWs (9.4%, 95% CI=7.2–11.6%). Regional differences in the pooled serological prevalence of SARS-CoV-2 IgG antibodies by gender and age groups is given in Fig. 3. It demonstrates that 12.7% (95% CI=7.12–18.31%) of male and 11.2% (95% CI=5.87–16.67%) of female HCWs in the USA were seropositive. Moreover, in the European zone, 8.6% (95% CI=6.3–10.9%) of male and 6.7% (95% CI=4.8–8.7%) of female HCWs had IgG antibodies. In East Asia, differences in the seroprevalence of HCWs by gender was found negligible.

Based on the work type of the HCWs, the high-risk group was found to have a seroprevalence of 11.9% (95% CI=8.4%−15.4) while intermediate- or low-risk HCWs had 8.6% (95% CI=6%−11.2%). HCWs from high and moderate-risk countries were observed to have a high seroprevalence of IgG antibodies relative to low-risk countries (Table 2).

In the three regions, the study focused on whether the participants' average or median age showed heterogeneous prevalence. In Europe, about 8.5% (95% CI=6.2–10.8%) of HCWs aged 40 years or older were IgG-positive compared to 4.8% (95% CI=1.5–8.0%) of HCWs aged less than 40 years. The difference between the age-groups was greater in East Asia, estimating an 8.53% (95% CI=0.3–24.5%) IgG seroprevalence among older (≥40 years of age) HCWs, while only 1.6% (95% CI=0.4–2.8%) of the young were infected. In contrast, in the United States, the pooled prevalence of IgG antibodies developed in the young HCWs was 17.7% (95% CI=9.5–26.0%) which was almost double compared to the older group (9.5%, 95% CI=5.1–13.9%). We intended to compare the two age groups (less than 40 years or 40 years and above) and thus, we assumed symmetry in the age distribution of each sample, which allowed us to take the mean and median age simultaneously in the analysis.

### 3.5 Sensitivity analyses for seroprevalence

We conducted sensitivity analyses that included each of the following types of studies: studies with more than 500 HCWs, regional differences from the studies with more than 500 HCWs, testing conducted with ELISA and pre-print studies. Forest plots are reported in Supplementary File 4. When considering studies of more than 500 participants, the overall seroprevalence among HCWs tends to be 8.02% (95% CI=6.23–9.82%). The seroprevalence was 9.3% (95% CI=5.5–13.2%) considering studies that conducted testing with ELISA. We also found the regional trend of seroprevalence between Europe, the USA, and East Asia was similar to the original studies after including studies that considered at least 500 HCWs. Moreover, we analyzed frequencies to estimate proportions from the 18 pre-print studies. The results from the supplements provide the seroprevalence was 8.0% (95% CI=5.6–10.4%). The findings are, therefore, similar to the meta-analysis of 53 studies.

### 3.6 Factors associated with SARS-CoV-2 IGG antibodies positivity

We investigated the associated factors for SARS-CoV-2 IgG sero-positive status by gender, age-group, country-wise risk and work-place risk of HCWs. Forest plots are given in Supplementary File 5. The overall pooled odds ratio of 25 studies for the association between gender and IgG antibody status was 1.18 (OR=1.18, 95% CI= 1.06–1.31) indicating the odds of catching an infection in male HCWs was higher by 18% than female. In most research, the prevalence of IgG antibodies per age-group was absent. We combined four studies to compare HCWs below the age of 50 and HCWs at the age of 50 and above. The odds between these two age groups were not significantly different (OR=1.09, 95% CI=0.67–1.77, reference: 50 years and over). In addition, we observed that high-risk HCWs were 1.62 times more likely to develop IgG antibodies than low or intermediate-risk HCWs, indicating that high-risk HCWs were 62% more at risk of infection than low or intermediate-risk HCWs (OR=1.62, 95% CI=1.04–2.58).

## 4 Discussion

This study investigated serum SARS-CoV-2 IgG antibody status of 173,353 HCWs of 14 countries obtained from 53 studies, which could help explain vaccine seroconversion effectiveness. Based on reported antibody findings, we investigated the variations in pooled seroprevalence of Europe, the USA, and East Asia.

Many national and regional studies have performed to estimate the seroprevalence of SARS-CoV-2 IgG antibodies in the general population [19], [20], [21], [22]. In a meta-analysis, SARS-CoV-2 seroprevalence ranged from 0.37% to 22.1% for the general population and found a pooled estimate of 3.38% [19]. Another meta-analysis of 338 studies involving 2.3 million individuals from 50 countries found that in the general population, SARS-CoV-2 antibody seroprevalence was as low as 3.2% [20]. Studies reported HCWs to suffer a significant risk from COVID-19, with the most vulnerable population being those employed in hospital environments [23], [24], [25], [26]. Our study calculated the pooled seroprevalence of SARS-CoV-2 IgG antibodies among the HCWs 8.6%, which is higher than the general population. Similar meta researches find different seroprevalences in the HCWs, varying from 7% to 11% [15], [16], [17].

The differences in the future precautions taken against the virus could be based on the regional variations of seroprevalence of SARS-CoV-2 IgG antibodies. It appears from our analysis that seroprevalence was higher in studies that were conducted in the USA compared to those in Europe and East Asia. The result is consistent with a meta-analysis that found that the proportion of SARS-CoV-2-positive HCWs was about a one-third of all COVID-19 patients of China compared to the USA and a half to Europe [16]. This reflects the strong adherence of HCWs in East Asia to infection prevention and control measures and the appropriate use of personal protective equipment's. The USA also seemed unprepared to cope with the surge in patients that led to a severe shortage of personal protective equipment leading to increased number of cases at the health care centers. [27].

Moreover, our pooled estimates indicate that younger HCWs were infected more compared to older HCWs in the United States. At the early stage of pandemic, an analysis of cases by the Center for Disease Control and Prevention from the USA revealed that 38% of those who were ill enough to be treated in hospital were younger than 55 [28]. It depicts that the virus might not be taken seriously by younger generations of the USA [29]. The US data also showed a dramatic rise in cases among the under-40 age-group who perceived themselves as less likely to contract a serious case of illness, and such second-wave behavior had let their guard down [30]. In our meta-analysis, the scenario was opposite in the case of Europe and East Asia where the elderly HCWs exhibited a higher prevalence of seropositive IgG antibodies than the USA. All except one (Japan) of the top 30 countries with the highest number of older citizens are from Europe and thus were affected most by the pandemic [31]. This partly explains why higher number of older HCWs developed IgG antibodies compared to younger group in Europe. Further analysis did not indicate a significant association between the age-group association with serum SARS-CoV-2 IgG antibody positivity. However, since we combined only four studies to measure the pooled odds ratio, the relationship between age and infection requires further investigation.

We also found the HCWs who worked in inpatient settings had a high prevalence of IgG antibodies. Studies also found that compared with the low or intermediate risk of HCWs, there was an increased risk of transmission in all health care settings for front-line HCWs [22]. The odds ratio also suggests a significant association between high-risk HCWs and catching infection. This highlights the importance of ensuring the availability of the patient care equipment and other aspects of following hospital safety protocols, including proper application and removal of the PPE. Thus, the likelihood of HCWs contributing to the spread of infections to the community is high, particularly when they are asymptomatic or mildly symptomatic.

Over time, we observed an increase in seroprevalence from about 5% in February-April to about 10% in May-September, which was anticipated for seroconversion in given time. The findings also resonate with the expectation that, relative to low-infection level countries, most antibodies were produced in HCWs from high-risk countries. Community transmission thus played a crucial role in the data on seroprevalence.

It is also evident from our study that male HCWs had higher pooled prevalence of serum IgG antibodies against SARS-CoV-2 than the females. With few exceptions, the gender bias observed in COVID-19 infection is a worldwide phenomenon. Researches published demonstrates similar trend, nevertheless, they also indicate no statistically significant difference in the prevalence of antibodies by gender similar to our analysis when observed closely. [20,29,32] Gender differences have been previously studied in adaptive immune systems and may account for the female advantage in COVID-19 [30]. This explains why such difference in circulating antibodies is not significant, though males were more prone to be infected than females.

In our meta-analysis, high heterogeneity suggests variation in study outcomes between the included studies. The heterogeneity was not fully explained by geographical region, gender, age-group, workplace infection risk, or country-wise infection risk. We speculate that there may be heterogeneity within the population, caused by other variables such as socioeconomic status, lifestyle, culture, and hospital protocol coverage. It could be argued that the high heterogeneity across the included studies could render the estimates of pooled prevalence less useful; however, high heterogeneity may also suggest that there is a large variation in the seroprevalence of pre-existing IgG antibodies across geogrphic regions, gender, age groups and country-wise risk level of infection.

In this study, the results are subjected to at least three limitations. First, the heterogeneity was very high across studies. However, to resolve this constraint, we conducted a random-effects model and subgroup analysis. Second, depending on the antibody tests applied, the seroprevalence reported in studies may be under or overestimated. The validity (sensitivity and specificity) of the antibody tests in most of the included studies has not been published. Third, many of the cross-sectional seroprevalence studies included in the meta-analysis aimed to evaluate immunity and were likely to underestimate the previous infection rates because antibodies tend to be detectable for a discrete period after infection.

This study showed an overall small proportion of HCWs from East Asia developed SARS-CoV-2 IgG antibodies. The high seroprevalence of antibodies in the United States suggests that the country has the most substantial evidence of challenges in high-risk countries. Herd immunity theory due to acute exposure to infection is questionable due to the slow progression of seroprevalence worldwide, and vaccination attempts to develop antibodies could be useful. Balanced resource allocation for East Asian, the US, and European countries should be considered to halt disease transmission, especially in male HCWs and increasing age. This outcome may have important implications for prioritizing vaccines' delivery and investigating the time needed by geographical regions for achieving herd immunity. Also, this study with study period evaluation gives us an understanding of the slow progression of long-term immunity against SARS-CoV-2.

## Contributors

AH, and SMN contributed to the literature search and study concept and design. AH, SMN, ZN, MKH, and MMH contributed to the data acquisition. AH accessed the data and contributed to the data analysis. AH, SMN and ZN contributed to the data interpretation. AH, MKH and MMH drafted the manuscript. All authors contributed to critical revision to the manuscript.

## Data sharing

Because this meta­ analysis was based on data extracted from previously published research, most of the data and study materials are available in the public domain. For further discussions, we invite interested parties to contact the corresponding author.

## Funding

None.

## Declaration of Competing Interest

All other authors declare no competing interests.

## Figures and Tables

**Table:** 

**A. PubMed**		
#1	(“Seroprevalence” OR “Prevalence” OR “Proportion”) AND (“Antibody” OR “Antibodies” OR “IgG” OR “IgM” OR “Immunoglobulins” OR “Immunoglobulin”)	81,768
#2	("COVID-19″ OR "Coronavirus disease" OR "Coronavirus" OR "SARS-CoV-2″) AND ("Healthcare workers")	3377
#3	#1 AND #2	43
Searching date starting from 01/01/2020 to 15/01/2021 All the entries were under ‘All Fields’ category		
**B. Google Scholar**		
#1	(“Seroprevalence” OR “Prevalence” OR “Proportion”) AND (“Antibody” OR “Antibodies” OR “IgG” OR “IgM” OR “Immunoglobulins” OR “Immunoglobulin”)	46,000
#2	("COVID-19″ OR "Coronavirus disease" OR "Coronavirus" OR "SARS-CoV-2″) AND ("Healthcare workers")	19,000
#3	#1 AND #2	30
Searching date starting from 01/01/2020 to 15/01/2021 All the searches were filtered to find articles published in 2020 only		
**C. ScienceDirect**		
#1	(“Seroprevalence” OR “Prevalence”) AND (“Antibodies” OR “IgG” OR “IgM” OR “Immunoglobulins”)	13,847
#2	("COVID-19″ OR "SARS-CoV-2″) AND ("Healthcare workers")	2175
#3	#1 AND #2	95
Searching date starting from 01/01/2020 to 15/01/2021 All the searches were filtered to find articles with study period 2020 only		

## Appendix B Supplementary materials

Image, application 1

Image, application 2

Image, application 3

Image, application 4

Image, application 5

Image, application 6
