# Supplementary material for: Seroprevalence of SARS-CoV-2 IgG antibodies among health care workers prior to vaccine administration in Europe, the USA and East Asia: A systematic review and meta-analysis
Source: eClinicalMedicine. 2021 Mar 8;33:100770. doi: 10.1016/j.eclinm.2021.100770 (PMC7938754; doi:10.1016/j.eclinm.2021.100770)
Supplement: Supplementary file 1 [file mmc1.docx]

**Supplementary file 1:** S1. Study quality assessment details for cohort, and cross-sectional studies by New-Castle Ottawa Scale.

**Supplementary File 1 Quality Assessment of Included Studies by New-Castle Ottawa Scale (Adapted)**

| **Cohort study** | | | | |
| --- | --- | --- | --- | --- |
| **Study** | **Selection** | **Comparability** | **Exposure/Outcome** | **Score** |
| **Herzberg et al.** [51] | ★★★ | ★ | ★★★ | 7 |
| **Iversen et al.** [32] | ★★★ | ★ | ★★★ | 7 |
| **Kohler et al.** [55] | ★★★ | ★ | ★★ | 6 |
| **Moscola et al.** [64] | ★★★ | ★ | ★★★ | 7 |
| **Pallett et al.** [66] | ★★★ | ★ | ★★ | 6 |

| **Cross-sectional Study** | | | | |
| --- | --- | --- | --- | --- |
| **Study** | **Selection** | **Comparability** | **Exposure** | **Score** |
| **Amendola et al.** [40] | ★★★★ | - | ★★★ | 7 |
| **Bampoe et al.** [41] | ★★★ | - | ★★★ | 6 |
| **Barallat et al.** [42] | ★★★ |  | ★★ | 5 |
| **Black et al.** [42] | ★★★ | - | ★★ | 5 |
| **Blairon et al.** [43] | ★★★★ | - | ★★ | 6 |
| **Brant-Zawadski et al.** [44] | ★★★ | - | ★★ | 5 |
| **Brunner et al.** [45] | ★★★★ | - | ★★★ | 7 |
| **Chen et al.** [46] | ★★★★ | - | ★★★ | 7 |
| **Corradini et al.** [47] | ★★★★ | - | ★★★ | 7 |
| **D. Sims et al.** [48] | ★★★ | - | ★★★ | 6 |
| **Dacosta-Urbieta et al.** [49] | ★★★ | - | ★★ | 5 |
| **Delmas et al.** [50] | ★★★★ | - | ★★★ | 7 |
| **Duysburgh et al.** [51] | ★★★ | - | ★★★ | 6 |
| **Fernandez et al.** [52] | ★★★ | - | ★★ | 5 |
| **Fujita et al.** [53] | ★★★ | - | ★★ | 5 |
| **Godbout et al.** | ★★★★ | - | ★★★ | 7 |
| **Hibino et al.** [52] | ★★★★ | - | ★★★ | 7 |
| **Hunter et al.** [30] | ★★★★ | - | ★★★ | 7 |
| **Iversen et al.** [32] | ★★★ | - | ★★★ | 6 |
| **Jeremias et al.** [53] | ★★★★ | - | ★★★ | 7 |
| **Khalil et al.** [54] | ★★★ | - | ★★★ | 6 |
| **Ko et al.** [57] | ★★★ | - | ★★★ | 6 |
| **Korth et al.** [56] | ★★★★ | - | ★★★ | 7 |
| **Lackermair et al.** [57] | ★★★ | - | ★★ | 5 |
| **Lahner et al.** [58] | ★★★ | - | ★★ | 5 |
| **Lindahl et al.** [59] | ★★★ | - | ★★★ | 6 |
| **Lidstrom et al.** [60] | ★★★ | - | ★★★ | 6 |
| **Madsen et al.** [61] | ★★★★ | - | ★ | 5 |
| **Mansour et al.** [62] | ★★★ | - | ★★★ | 6 |
| **Martin et al.** [63] | ★★★ | - | ★★★ | 6 |
| **Olalla et al.** [65] | ★★★★ | - | ★★★ | 7 |
| **Piccoli et al.** [67] | ★★★★ | - | ★★★ | 7 |
| **Plebani et al.** [68] | ★★★★ | - | ★★★ | 7 |
| **Poulikakos et al.** [69] | ★★★ | - | ★★ | 5 |
| **Psichogiou et al.** [36] | ★★★★ | - | ★★ | 6 |
| **Rudberg et al.** [17] | ★★★★ | - | ★★★ | 7 |
| **Schmidt et al.** [70] | ★★★ | - | ★★★ | 6 |
| **Solodky et al.** [71] | ★★★ | - | ★★ | 5 |
| **Sotgiu et al.** [72] | ★★★★ | - | ★★★ | 7 |
| **Steensels et al.** [73] | ★★★★ | - | ★★★ | 7 |
| **Stock et al.** [74] | ★★★★ | - | ★★★ | 7 |
| **Stubblefield et al.** [75] | ★★★ | - | ★★★ | 6 |
| **Sydney et al.** [76] | ★★★ | - | ★★ | 5 |
| **Takita et al.** [77] | ★★★★ | - | ★★★ | 7 |
| **Tu et al.** [78] | ★★★★ | - | ★★★ | 7 |
| **Varona et al.** [81] | ★★★ | - | ★★ | 5 |
| **Venugopal et al.** [79] | ★★★ | - | ★★★ | 6 |
| **Vlachoyiannopoulos et al.** [80] | ★★★ | - | ★★ | 5 |
| **Xin Xu et al.** [84] | ★★★★ | - | ★★ | 6 |

**Adapted Newcastle Ottawa Quality Assessment Scale**

**Cohort Studies [1, 2]**

Note: A study can be awarded a maximum of one point for each numbered item with the Selection and Exposure categories. A maximum of two points can be given for Comparability.

**Selection (maximum 4)**

**1.** Representativeness of the cohort

a. Truly representative of the average in the target population (random sample or whole)*

b. Somewhat representative of the average in the target population (purposive sampling of representative hospital or healthcare facilities or evidence that the sample is representative of the source population)*

c. Selected group of users/convenient sampling

d. No description of the derivation of the cohort

**2.** Selection of the non-exposed cohort

a. Drawn from the same community as the exposed cohort or hospitals serving the same population as cohort *

b. Drawn from a different source

c. No description of the derivation of the non-exposed cohort

**3.** Ascertainment of exposure

a. Requires some independent validation (in which diagnostic method it was ascertained)*

b. Clinical/Hospital/Medical record*

c. Self-reported with no reference to primary record

d. No description

**4.** Demonstration that outcome of interest (Multidrug resistant tuberculosis) was accounted for or not present at start of study

a. Yes *

b. No

**Comparability (maximum 2)**

**1.** Comparability of cohorts on the basis of the design or analysis

a. Study controls for the variable: Previous tuberculosis treatment history*

b. Study controls for any additional socio-demographic factor (e.g. age, sex etc.) *

**Outcome (maximum 3)**

**1.** Ascertainment of outcome (Seroprevalence of anti-COVID IgG)

a. Requires some independent validation (in which diagnostic method it was ascertained)*

b. Clinical/Hospital/Medical record*

c. Self-reported with no reference to primary record

d. No description

**2.** Was follow-up long enough for outcomes to occur?

a. Yes (>1 year)*

b. No

**3.** Adequacy of follow-up of cohorts

a. Complete follow-up – all subjects accounted for *

b. Subjects lost to follow-up unlikely to introduce bias – small number lost (<20%) or attrition described and accounted for in analysis *

c. Follow up rate not adequate and no description of those lost

d. No statement

**Cross-Sectional Studies [3]**

**Selection (maximum 5)**

**1.** Representativeness of the sample

a. Truly representative of the average in the target population (random sample or whole population) *

b. Somewhat representative of the average in the target population (purposive sampling of representative schools or evidence that the sample is representative of the source population) *

c. Selected group of users/convenience sampling

d. No description of the sampling strategy

**2.** Sample size

a. Justified and satisfactory*

b. Adequately powered to detect a difference (at least 10 events per variable in multivariate analyses)*

c. Not justified

**3.** Non-respondents

a. Comparability between respondents and non-respondents characteristics is established, and the response rate is satisfactory (>80%)*

b. The response rate is unsatisfactory, or the comparability between respondents and non-respondents is unsatisfactory

c. No description of the response rate or the characteristics of the responders & non-responders

**4.** Ascertainment of the exposure

a. Requires some independent validation (in which diagnostic method it was ascertained)**

b. Clinical/Hospital/Medical record*

c. Self-reported with no reference to primary record

d. No description

**Comparability (maximum 2)**

**1.** The subjects in different outcome groups are comparable, based on the study design or analysis. Confounding factors are controlled

a. Study controls for the variable: Previous tuberculosis treatment history*

b. Study controls for any additional socio-demographic factor (e.g. age, sex etc.) *

**Outcome (maximum 3)**

**1.** Assessment of the outcome (Seroprevalence of anti-COVID IgG)

a. Requires some independent validation (in which diagnostic method it was ascertained)**

b. Clinical/Hospital/Medical record*

c. Self-reported with no reference to primary record

d. No description

**2.** Statistical test

a. The statistical test used to analyze the data is clearly described and appropriate, and the measurement of the association is presented as either an OR, CI and P value or a beta coefficient, SE and P value*

b. The statistical test is not appropriate, not described or incomplete

**Adapted from previous published literature:**

1. Ottawa Hospital Research Institute. http://www.ohri.ca/programs/clinical_epidemiology/oxford.asp (accessed Oct 23, 2019).
2. Epstein S, Roberts E, Sedgwick R, *et al.* Poor school attendance and exclusion: a systematic review protocol on educational risk factors for self-harm and suicidal behaviours. *BMJ Open* 2018; **8**: e023953.
3. Herzog R, Álvarez-Pasquin MJ, Díaz C, Del Barrio JL, Estrada JM, Gil Á. Are healthcare workers’ intentions to vaccinate related to their knowledge, beliefs and attitudes? A systematic review. *BMC Public Health* 2013; **13**: 154.
