# Supplementary material for: Seroprevalence of SARS-CoV-2 IgG antibodies among health care workers prior to vaccine administration in Europe, the USA and East Asia: A systematic review and meta-analysis
Source: eClinicalMedicine. 2021 Mar 8;33:100770. doi: 10.1016/j.eclinm.2021.100770 (PMC7938754; doi:10.1016/j.eclinm.2021.100770)
Supplement: Supplementary file 3 [file mmc3.docx]

**Supplementary file 3:** References of studies that were excluded after full-text review.

**Excluded Articles References:**

1. Abo-Leyah, H., Gallant, S., Cassidy, D., Giam, Y. H., Killick, J., Marshall, B., Hay, G., Pembridge, T., Strachan, R., Gallant, N., Parcell, B. J., George, J., Furrie, E., & Chalmers, J. D. (2020). Seroprevalence of SARS-COV-2 Antibodies in Scottish Healthcare Workers. *MedRxiv*. https://doi.org/10.1101/2020.10.02.20205641

2. Alajmi, J., Jeremijenko, A. M., Abraham, J. C., Alishaq, M., Concepcion, E. G., Butt, A. A., & Abou-Samra, A. B. (2020). COVID-19 infection among healthcare workers in a national healthcare system: The Qatar experience. *International Journal of Infectious Diseases*, *100*, 386–389. https://doi.org/10.1016/j.ijid.2020.09.027

3. Alharbi, S. A., Almutairi, A. Z., Jan, A. A., & Alkhalify, A. M. (2020). Enzyme-Linked Immunosorbent Assay for the Detection of Severe Acute Respiratory Syndrome Coronavirus 2 (SARS-CoV-2) IgM/IgA and IgG Antibodies Among Healthcare Workers. *Cureus*, *12*(9). https://doi.org/10.7759/cureus.10285

4. Alkurt, G., Murt, A., Aydin, Z., Tatli, O., Bugra AGAOGLU, N., Aydin, M., Karaali, R., Gunes, M., Yesilyurt, B., Mardinoglu, A., Doganay, M., Basinoglu, F., Dinler Doganay, G., Doganay, L., Farabi Egitim ve Arastirma Hastanesi, D., & Biyokimya Bolumu, T. (2020). from Three Pandemic Hospitals of Turkey 2. *Health Institutes of Turkey (TUSEB)*, *34718*, 2020.08.19.20178095. https://doi.org/10.1101/2020.08.19.20178095

5. Asuquo, M. I., Effa, E., Otu, A., Ita, O., Udoh, U., Umoh, V., Gbotosho, O., Ikpeme, A., Ameh, S., Egbe, W., Etok, M., Guck, J., & Ekpenyong, A. (2020). Prevalence of IgG and IgM antibodies to SARS-CoV-2 among clinic staff and patients. *MedRxiv*, 2020.07.02.20145441. https://doi.org/10.1101/2020.07.02.20145441

6. Bahrs, C., Kimmig, A., Weis, S., Ankert, J., Hagel, S., Stallmach, A., Steiner, A., Bauer, M., Behringer, W., Baier, M., Kesselmeier, M., Richert, C., Zepf, F., Walter, M., Kiehntopf, M., Löffler, B., & Pletz, M. W. (2020). Seroprevalence of SARS CoV-2 antibodies in healthcare workers and 1 administration employees: a prospective surveillance study at a 1,400-2 bed university hospital in Germany 3 4. *MedRxiv*, 2020.09.29.20203737. https://doi.org/10.1101/2020.09.29.20203737

7. Baracco, A., Perotti, G. M., Filippin, A., Anesi, A., Beccarini, V., Raimondi, L., Galvani, S., Bosio, D., & Bergamaschi, E. (2020). SARS-CoV-2 Antibody Prevalence in Health Care Workers of Lodi Hospital, the COVID-19 Italian Epicentre. *SSRN Electronic Journal*, 1–22. https://doi.org/10.2139/ssrn.3650227

8. Bryan, A., Pepper, G., Wener, M. H., Fink, S. L., Morishima, C., Chaudhary, A., Jerome, K. R., Mathias, P. C., & Greninger, A. L. (2020). Performance characteristics of the abbott architect sars-cov-2 igg assay and seroprevalence in Boise, Idaho. *Journal of Clinical Microbiology*, *58*(8). https://doi.org/10.1128/JCM.00941-20

9. Bhattacharyya, R., Bhaduri, R., Ritoban Kundu, ;, Salvatore, ; Maxwell, & Mukherjee, B. (2020). Reconciling epidemiological models with misclassified case-counts for SARS-CoV-2 with seroprevalence surveys: A case study in Delhi, India. *MedRxiv*. https://doi.org/10.1101/2020.07.31.20166249

10. Brandstetter, S., Roth, S., Harner, S., Buntrock-Döpke, H., Toncheva, A. A., Borchers, N., Gruber, R., Ambrosch, A., & Kabesch, M. (2020). Symptoms and immunoglobulin development in hospital staff exposed to a SARS-CoV-2 outbreak. *Pediatric Allergy and Immunology*, *31*(7), 841–847. https://doi.org/10.1111/pai.13278

11. Bruni, M., Cecatiello, V., Diaz-Basabe, A., Lattanzi, G., Mileti, E., Monzani, S., Pirovano, L., Rizzelli, F., Visintin, C., Bonizzi, G., Giani, M., Lavitrano, M., Faravelli, S., Forneris, F., Caprioli, F., Pelicci, P. G., Natoli, G., Pasqualato, S., Mapelli, M., & Facciotti, F. (2020). Persistence of Anti-SARS-CoV-2 Antibodies in Non-Hospitalized COVID-19 Convalescent Health Care Workers. *Journal of Clinical Medicine*, *9*(10), 3188. https://doi.org/10.3390/jcm9103188

12. Bryan, A., Pepper, G., Wener, M. H., Fink, S. L., Morishima, C., Chaudhary, A., Jerome, K. R., Mathias, P. C., & Greninger, A. L. (2020). Performance characteristics of the abbott architect sars-cov-2 igg assay and seroprevalence in Boise, Idaho. *Journal of Clinical Microbiology*, *58*(8). https://doi.org/10.1128/JCM.00941-20

13. Buntinx, F., Claes, P., Gulikers, M., Verbakel, J., De Lepeleire, J., Van der Elst, M., Van Ranst, M., & Vermeersch, P. (2020). Early experiences with antibody testing in a Flemish nursing home during an acute COVID-19 outbreak: a retrospective cohort study. *MedRxiv*, 2020.05.18.20105874. https://doi.org/10.1101/2020.05.18.20105874

14. Cervia, C., Nilsson, J., Zurbuchen, Y., Valaperti, A., Schreiner, J., Wolfensberger, A., Raeber, M., Adamo, S., Emmenegger, M., Hasler, S., Bosshard, P., De Cecco, E., Bächli, E., Rudiger, A., Stüssi-Helbling, M., Huber, L., Zinkernagel, A., Schaer, D., Aguzzi, A., … Boyman, O. (2020). Systemic and mucosal antibody secretion specific to SARS-CoV-2 during mild versus severe COVID-19. *BioRxiv*, 2020.05.21.108308. https://doi.org/10.1101/2020.05.21.108308

15. Chong, Y., Tani, N., Terazawa, N., Hospital, K., Nakashima, H., Shimono, N., & Tanaka, Y. (2020). *Genetic testing and serological screening for SARS-CoV-2 infection in a COVID-19 outbreak in a nursing facility in Japan*. https://doi.org/10.21203/rs.3.rs-64266/v1

16. Clarke, C., Prendecki, M., Dhutia, A., Ali, M. A., Sajjad, H., Shivakumar, O., Lightstone, L., Kelleher, P., Pickering, M. C., Thomas, D., Charif, R., Griffith, M., McAdoo, S. P., & Willicombe, M. (2020). High Prevalence of Asymptomatic COVID-19 Infection in Hemodialysis Patients Detected Using Serologic Screening. *Journal of the American Society of Nephrology*, *31*(9), 1969–1975. https://doi.org/10.1681/ASN.2020060827

17. Claudia, I., G, S., V, F., C, H., A, S., F, F., MF, O., & O, B. (2020). Seroprevalence of SARS-CoV-2 antibodies among physicians from a children’s hospital. *Archivos Argentinos de Pediatria*, *118*(6). https://doi.org/10.5546/aap.2020.eng.381

18. Comar, M., Brumat, M., Concas, M. P., Argentini, G., Bianco, A., Bicego, L., Bottega, R., Carli, P., Cassone, A., Catamo, E., Cocca, M., Del Pin, M., Di Stazio, M., Feresin, A., La Bianca, M., Morassut, S., Morgan, A., Pelliccione, G., Petix, V., … Gasparini, P. (2020). COVID-19 experience: first Italian survey on healthcare staff members from a Mother-Child Research hospital using combined molecular and rapid immunoassays test. *MedRxiv*, 2020.04.19.20071563. https://doi.org/10.1101/2020.04.19.20071563

19. Cooper, D. J., Lear, S., Watson, L., Shaw, A., Ferris, M., Doffinger, R., Sharrocks, K., Weekes, M. P., Warne, B., Sparkes, D., Jones, N. K., Routledge, M., Chaudhry, A., Dempsey, K., Matson, M., Lakha, A., Gathercole, G., Wilson, E., Shahzad, O., … Baker, S. (2020). A prospective study of risk factors associated with seroprevalence of SARS-CoV-2 antibodies in 1 healthcare workers at a large UK teaching hospital 2 3. *MedRxiv*, 2020.11.03.20220699. https://doi.org/10.1101/2020.11.03.20220699

20. Crovetto, F., Crispi, F., Llurba, E., Figueras, F., Gómez-Roig, M. D., & Gratacós, E. (2020). Seroprevalence and presentation of SARS-CoV-2 in pregnancy. *The Lancet*, *396*(10250), 530–531. https://doi.org/10.1016/S0140-6736(20)31714-1

21. Dimeglio, C., Herin, F., Miedougé, M., Cambus, J. P., Abravanel, F., Mansuy, J. M., Soulat, J. M., & Izopet, J. (2020). Screening for SARS-CoV-2 antibodies among healthcare workers in a university hospital in southern France. *Journal of Infection*. https://doi.org/10.1016/j.jinf.2020.09.035

22. Ebinger, J. E., Botwin, G. J., Albert, C. M., Alotaibi, M., Arditi, M., Berg, A. H., Binek, A., Botting, P., Fert-Bober, J., Figueiredo, J. C., Grein, J. D., Hasan, W., Henglin, M., Hussain, S. K., Jain, M., Joung, S., Karin, M., Kim, E. H., Li, D., … Cheng, S. (2020). SARS-CoV-2 Seroprevalence Across a Diverse Cohort of Healthcare Workers. *MedRxiv*, 2020.07.31.20163055. https://doi.org/10.1101/2020.07.31.20163055

23. Epstude, J., & Harsch, I. A. (2020). Seroprevalence of COVID-19 antibodies in the cleaning and oncological staff of a municipal clinic. *GMS Hygiene and Infection Control*, *15*, Doc18. https://doi.org/10.3205/dgkh000353

24. Erber, J., Kappler, V., Haller, B., Mijočević, H., Prazeres da Costa, C., Gebhardt, F., Graf, N., Hoffmann, D., Thaler, M., Lorenz, E., Roggendorf, H., Henkel, A., Menden, M. P., Ruland, J., Spinner, D., Protzer, U., Knolle, P., & Lingor, P. (2020). Strategies for infection control and prevalence of anti-SARS-CoV-2 IgG in 4,554 employees of a university hospital in Munich, Germany. *MedRxiv*, 2020.10.04.20206136. https://doi.org/10.1101/2020.10.04.20206136

25. Favara, D. M., Cooke, A., Doffinger, R., McAdam, K., Corrie, P., & Ainsworth, N. L. (2020). COVID-19 Serology in Oncology Staff Study: Understanding SARS-CoV-2 in the Oncology Workforce. In *Clinical Oncology* (Vol. 33, Issue 1, p. e61). Elsevier Ltd. https://doi.org/10.1016/j.clon.2020.07.015

26. Feehan, A., Fort, D., Garcia-Diaz, J., Price-Haywood, E., Velasco, C., Sapp, E., Pevey, D., & Seoane, L. (2020). Point prevalence of SARS-CoV-2 and infection fatality rate in Orleans and Jefferson Parish, Louisiana, May 9-15, 2020. *MedRxiv*, 2020.06.23.20138321. https://doi.org/10.1101/2020.06.23.20138321

27. Flower, B., Brown, J. C., Simmons, B., Moshe, M., Frise, R., Penn, R., Kugathasan, R., Petersen, C., Daunt, A., Ashby, D., Riley, S., Atchison, C. J., Taylor, G. P., Satkunarajah, S., Naar, L., Klaber, R., Badhan, A., Rosadas, C., Khan, M., … Cooke, G. S. (2020). Clinical and laboratory evaluation of SARS-CoV-2 lateral flow assays for use in a national COVID-19 seroprevalence survey. *Thorax*, *75*(12), 1082–1088. https://doi.org/10.1136/thoraxjnl-2020-215732

28. Fusco, F. M., Pisaturo, M., Iodice, V., Bellopede, R., Tambaro, O., Parrella, G., Di Flumeri, G., Viglietti, R., Pisapia, R., Carleo, M. A., Boccardi, M., Atripaldi, L., Chignoli, B., Maturo, N., Rescigno, C., Esposito, V., Dell’Aversano, R., Sangiovanni, V., & Punzi, R. (2020). COVID-19 among healthcare workers in a specialist infectious diseases setting in Naples, Southern Italy: results of a cross-sectional surveillance study. *Journal of Hospital Infection*, *105*(4), 596–600. https://doi.org/10.1016/j.jhin.2020.06.021

29. Galan, I., Velasco, M., Casas, M. L., Goyanes, M. J., Rodriguez-Caravaca, G., Losa, J., Noguera, C., & Castilla, V. (2020). SARS-CoV-2 SEROPREVALENCE AMONG ALL WORKERS IN A TEACHING HOSPITAL IN SPAIN: UNMASKING THE RISK. *MedRxiv*, 2020.05.29.20116731. https://doi.org/10.1101/2020.05.29.20116731

30. Garcia-Basteiro, A. L., Moncunill, G., Tortajada, M., Vidal, M., Guinovart, C., Jiménez, A., Santano, R., Sanz, S., Méndez, S., Llupià, A., Aguilar, R., Alonso, S., Barrios, D., Carolis, C., Cisteró, P., Chóliz, E., Cruz, A., Fochs, S., Jairoce, C., … Dobaño, C. (2020). Seroprevalence of antibodies against SARS-CoV-2 among health care workers in a large Spanish reference hospital. *Nature Communications*, *11*(1). https://doi.org/10.1038/s41467-020-17318-x

31. Goenka, M., Shah, B., Goenka, U., Das, S. S., Afzalpurkar, S., Mukherjee, M., Patil, V. U., Jajodia, S., Rodge, G., Khan, U., & Bandopadhyay, S. (2020). COVID-19 prevalence among health‐care workers of Gastroenterology department: An audit from a tertiary‐care hospital in India. *JGH Open*. https://doi.org/10.1002/jgh3.12447

32. Grant, J., Wilmore, S., McCann, N., Donnelly, O., Lai, R., Kinsella, M., Rochford, H., Patel, T., Kelsey, M., & Andrews, J. (2020). Seroprevalence of SARS-CoV-2 antibodies in healthcare workers at a London NHS Trust. *Infection Control and Hospital Epidemiology*. https://doi.org/10.1017/ice.2020.402

33. Guedez-López, G. V., Alguacil-Guillén, M., González-Donapetry, P., Bloise, I., Tornero-Marin, C., González-García, J., Mingorance, J., García-Rodríguez, J., Montero-Vega, M. D., Romero, M. P., García-Bujalance, S., Cendejas-Bueno, E., Ruiz-Carrascoso, G., Lázaro-Perona, F., Falces-Romero, I., Gutiérrez-Arroyo, A., Girón de Velasco-Sada, P., Rico Nieto, A., Loeches, B., … Molina Muñoz, E. (2020). Evaluation of three immunochromatographic tests for rapid detection of antibodies against SARS-CoV-2. *European Journal of Clinical Microbiology and Infectious Diseases*, *39*(12), 2289–2297. https://doi.org/10.1007/s10096-020-04010-7

34. Hains, D. S., Schwaderer, A. L., Carroll, A. E., Starr, M. C., Wilson, A. C., Amanat, F., & Krammer, F. (2020). Asymptomatic Seroconversion of Immunoglobulins to SARS-CoV-2 in a Pediatric Dialysis Unit. *JAMA - Journal of the American Medical Association*, *323*(23), 2424–2425. https://doi.org/10.1001/jama.2020.8438

35. Hamilton, F., Muir, P., Attwood, M., Vipond, A. N. B., Hopes, R., Moran, E., Maskell, N., Warwick, D., Albur, M., Turner, J., MacGowan, A., & Arnold, D. (2020). Kinetics and performance of the Abbott architect SARS-CoV-2 IgG antibody assay. *Journal of Infection*, *81*(6), e7. https://doi.org/10.1016/j.jinf.2020.07.031

36. Harsch, I. A., Skiba, M., Konturek, P. C., & Epstude, J. (2020). Prevalence of antibodies against COVID-19 in the staff of a COVID-19 regular ward. *GMS Hygiene and Infection Control*, *15*, Doc09. https://doi.org/10.3205/dgkh000344

37. Havers, F. P., Reed, C., Lim, T., Montgomery, J. M., Klena, J. D., Hall, A. J., Fry, A. M., Cannon, D. L., Chiang, C. F., Gibbons, A., Krapiunaya, I., Morales-Betoulle, M., Roguski, K., Rasheed, M. A. U., Freeman, B., Lester, S., Mills, L., Carroll, D. S., Owen, S. M., … Thornburg, N. J. (2020). Seroprevalence of Antibodies to SARS-CoV-2 in 10 Sites in the United States, March 23-May 12, 2020. *JAMA Internal Medicine*. https://doi.org/10.1001/jamainternmed.2020.4130

38. He, L., Zeng, Y., Zeng, C., Zhou, Y., Li, Y., Xie, X., Xu, W., Luo, W., Hu, J., Yi, Z., Wang, X., Tang, S., Xu, L., & Chen, C. (2020). Positive Rate of Serology and RT-PCR for COVID-19 among healthcare workers during different periods in Wuhan, China. *Journal of Infection*. https://doi.org/10.1016/j.jinf.2020.08.027

39. Houlihan, C., Vora, N., Byrne, T., Lewer, D., Heaney, J., Moore, D., Matthews, R., Adam, S., Enfield, L., Severn, A., McBride, A., Spyer, M. J., Beale, R., Cherepanov, P., Gaertner, K., Shahmanesh, M., Ng, K., Cornish, G., Walker, N., … Nastouli, E. (2020). SARS-CoV-2 virus and antibodies in front-line Health Care Workers in an acute hospital in London: preliminary results from a longitudinal study. *MedRxiv*, 2020.06.08.20120584. https://doi.org/10.1101/2020.06.08.20120584

40. Javed, W., Bin Baqar, J., Hussain, S., Abidi, B., & Farooq, W. (2020). Sero-prevalence Findings from Metropoles in Pakistan: Implications for Assessing COVID-19 Prevalence and Case-fatality within a Dense, Urban Working Population. *MedRxiv*. https://doi.org/10.1101/2020.08.13.20173914

41. Jeong, J. M., Radeos, M. S., Shee, B., Kindschuh, M., Hernandez, C., Sasson, C., Braciale, T. J., Freeze, M., & Kindschuh, W. (2020). COVID-19 Seroconversion in Emergency Professionals at an Urban Academic Emergency Department in New York City. In *Annals of Emergency Medicine* (Vol. 76, Issue 6, pp. 815–816). Mosby Inc. https://doi.org/10.1016/j.annemergmed.2020.06.038

42. Jerkovic, I., Ljubic, T., Basic, Z., Kruzic, I., Kunac, N., Bezic, J., Vuko, A., Markotic, A., & Andjelinovic, S. (2020). SARS-CoV-2 antibody seroprevalence in industry workers in Split-Dalmatia and Sibenik-Knin County, Croatia. *Journal of Occupational & Environmental Medicine*, 2020.05.11.20095158. https://doi.org/10.1101/2020.05.11.20095158

43. Jespersen, S., Mikkelsen, S., Greve, T., Kaspersen, K. A., Tolstrup, M., Kjaergaard Boldsen, J., Redder, J. D., Nielsen, K., Abildgaard, A. M., Kolstad, H. A., Østergaard, L., Thomsen, M. K., Møller, J., & Erikstrup, C. (2020). SARS-CoV-2 seroprevalence survey among 18,000 healthcare and administrative personnel at hospitals, pre-hospital services, and specialist practitioners in the Central Denmark Region. *MedRxiv*, 2020.08.10.20171850. https://doi.org/10.1101/2020.08.10.20171850

44. KAMMON, A., El-Arabi, A., Erhouma, E., Mehemed, T., & Mohamed, O. (2020). Seroprevalence of antibodies against SARS-CoV-2 among public community and health-care workers in Alzintan City of Libya. *MedRxiv*, 2020.05.25.20109470. https://doi.org/10.1101/2020.05.25.20109470

45. Kassem, A. M., Talaat, H., Shawky, S., Fouad, R., Amer, K., Elnagdy, T., Hassan, W. A., Tantawi, O., Abdelmoniem, R., Gaber, Y., Badary, H. A., & Musa, S. (2020). SARS-CoV-2 infection among healthcare workers of a gastroenterological service in a tertiary care facility. *Arab Journal of Gastroenterology*, *21*(3), 151–155. https://doi.org/10.1016/j.ajg.2020.07.005

46. Kontou, P. I., Braliou, G. G., Dimou, N. L., Nikolopoulos, G., & Bagos, P. G. (2020). Antibody tests in detecting SARS-CoV-2 infection: A meta-analysis. *Diagnostics*, *10*(5). https://doi.org/10.3390/diagnostics10050319

47. Kumar, D., Ferreira, V. H., Chruscinski, A., Kulasingam, V., Pugh, T. J., Dus, T., Mn, R. N., Wouters, B., Oza, A., Ierullo Msc, M., Ku Msc, T., Majchrzak-Kita, B., Humar, S. T., Bahinskaya Msc, I., Pinzon Msc, N., Zhang Phd, J., Heisler, L. E., Krzyzanowski, P. M., Lam, B., … Humar, A. (2020). Prospective Observational Study of Screening Asymptomatic Healthcare Workers for SARS-CoV-2 at a Canadian Tertiary Care Center. *MedRxiv*. https://doi.org/10.1101/2020.07.21.20159053

48. Ladhani, S. N., Jeffery-Smith, A. J., Patel, M., Janarthanan, R., Fok, J., Crawley-Boevey, E., Vusirikala, A., Fernandez, E., Sanchez-Perez, M., Tang, S., Dun-Campbell, K., Wynne-Evans, E., Bell, A., Patel, B., Amin-Chowdhury, Z., Aiano, F., Paranthaman, K., Ma, T., Saavedra-Campos, M., … Zambon, M. (2020). High prevalence of SARS-CoV-2 antibodies in care homes affected by COVID-19; a prospective cohort study in England. *MedRxiv*. https://doi.org/10.1101/2020.08.10.20171413

49. Lanman, R., & Lanman, T. (2020). *SARS-CoV-2 Serology Results in the First COVID-19 Case in California: A Case Report and Recommendations for Serology Testing and Interpretation*. https://doi.org/10.21203/rs.3.rs-35358/v1

50. Laursen, J., Petersen, J., Didriksen, M., Iversen, K., & Ullum, H. (2020). Prevalence of SARS-CoV-2 IgG/IgM antibodies among Danish and Swedish Falck emergency and non-emergency healthcare workers. *MedRxiv*. https://doi.org/10.1101/2020.09.26.20202259

51. Liu, T., Wu, S., Tao, H., Zeng, G., Zhou, F., Guo, F., & Wang, X. (2020). Prevalence of IgG antibodies to SARS-CoV-2 in Wuhan - implications for the ability to produce long-lasting protective antibodies against SARS-CoV-2. *MedRxiv*, 2020.06.13.20130252. https://doi.org/10.1101/2020.06.13.20130252

52. Lu, L., Zhang, H., Zhan, M., Jiang, J., Yin, H., Dauphars, D. J., Li, S.-Y., Li, Y., & He, Y.-W. (2020). *Antibody Response and Therapy in COVID-19 Patients: Significance in Vaccine Development*. https://doi.org/10.20944/preprints202008.0166.v1

53. Lumley, S. F., Wei, J., O’Donnell, D., Stoesser, N. E., Matthews, P. C., Howarth, A., Hatch, S. B., Marsden, B. D., Cox, S., James, T., Peck, L., Ritter, T., Toledo, Z. de, Cornall, R. J., Jones, E. Y., Stuart, D. I., Screaton, G., Ebner, D., Hoosdally, S., … Eyre, D. W. (2020). The duration, dynamics and determinants of SARS-CoV-2 antibody responses in individual healthcare workers. *MedRxiv*, 2020.11.02.20224824. https://doi.org/10.1101/2020.11.02.20224824

54. Malickova, K., Kratka, Z., Luxova, S., Bortlik, M., & Lukas, M. (2020). Anti-SARS-CoV-2 antibody testing in IBD healthcare professionals: are we currently able to provide COVID-free IBD clinics? *Scandinavian Journal of Gastroenterology*, *55*(8), 1–3. https://doi.org/10.1080/00365521.2020.1791244

55. Marshall, D., Bois, F., Jensen, S., Linde, S., Higby, R., Remy-McCort, Y., Murray, S., Dieckelman, B., & Sudradjat, F. (2020). Sentinel Coronavirus Environmental Monitoring Can Contribute to Detecting Asymptomatic SARS-CoV-2 Virus Spreaders and Can Verify Effectiveness of Workplace COVID-19 Controls. *Microbial Risk Analysis*, 2020.06.24.20131185. https://doi.org/10.1101/2020.06.24.20131185

56. Martín, V., Fernández-Villa, T., Lamuedra Gil de Gomez, M., Mencía-Ares, O., Rivero Rodríguez, A., Reguero Celada, S., Montoro Gómez, M., Nuevo Guisado, M. T., Villa Aller, C., Díez Flecha, C., Carvajal, A., & Fernández Vázquez, J. P. (2020). Prevalence of SARS-CoV-2 infection in general practitioners and nurses in primary care and nursing homes in the Healthcare Area of León and associated factors. *Semergen*, *46*(S1), 35–39. https://doi.org/10.1016/j.semerg.2020.05.014

57. Mesnil, M., Joubel, K., Yavchitz, A., Miklaszewski, N., & Devys, J. M. (2020). Seroprevalence of SARS-Cov-2 in 646 professionals at the Rothschild Foundation Hospital (ProSeCoV study). *Anaesthesia Critical Care and Pain Medicine*, *39*(5), 595–596. https://doi.org/10.1016/j.accpm.2020.08.003

58. MG, C., KC, J., R, K., J, M., V, K.-P., D, T., N, M., S, M., S, S., A, L., L, S., P, M., I, K., RL, B., A, M., B, M., C, P., J, M., HC, M., … KC, J. (2020). High SARS-CoV-2 seroprevalence in Health Care Workers but relatively low numbers of deaths in urban Malawi. *MedRxiv : The Preprint Server for Health Sciences*. https://doi.org/10.1101/2020.07.30.20164970

59. Moncunill, G., Mayor, A., Santano, R., Jiménez, A., Vidal, M., Tortajada, M., Sanz, S., Méndez, S., Llupià, A., Aguilar, R., Alonso, S., Barrios, D., Carolis, C., Cisteró, P., Chóliz, E., Cruz, A., Fochs, S., Jairoce, C., Hecht, J., … Garcia-Basteiro, A. L. (2020). SARS-CoV-2 infections and antibody responses among health care workers in a Spanish hospital after a month of follow-up. *MedRxiv*, 2020.08.23.20180125. https://doi.org/10.1101/2020.08.23.20180125

60. Morcuende, M., Guglielminotti, J., & Landau, R. (2020). Anesthesiologists’ and intensive care providers’ exposure to COVID-19 infection in a new york city academic center: A prospective cohort study assessing symptoms and COVID-19 antibody testing. *Anesthesia and Analgesia*, *131*(3), 669–676. https://doi.org/10.1213/ANE.0000000000005056

61. Mughal, M. S., Kaur, I. P., Patton, C. D., Mikhail, N. H., Vareechon, C., & Granet, K. M. (2020). The Prevalence of SARS-CoV-2 IgG Antibodies in Intensive Care Unit (ICU) Healthcare Personnel (HCP) and its Implications - A Single-Center, Prospective, Pilot Study. *Infection Control and Hospital Epidemiology*, 1. https://doi.org/10.1017/ice.2020.298

62. Nakamura, A., Sato, R., Ando, S., Oana, N., Nozaki, E., Endo, H., Miyate, Y., Soma, J., & Miyata, G. (2020). Seroprevalence of Antibodies to SARS-CoV-2 in Healthcare Workers in Non-epidemic Region: A Hospital Report in Iwate Prefecture, Japan. *MedRxiv*, 2020.06.15.20132316. https://doi.org/10.1101/2020.06.15.20132316

63. Nopsopon, T., Pongpirul, K., Chotirosniramit, K., & Hiransuthikul, N. (2020). COVID-19 Antibody in Thai Community Hospitals. *MedRxiv*, 2020.06.24.20139188. https://doi.org/10.1101/2020.06.24.20139188

64. Pancrazzi, A., Magliocca, P., Lorubbio, M., Vaggelli, G., Galano, A., Mafucci, M., Duranti, D., Cortesi, M., Mazzeschi, E., Fabbroni, S., Viti, G., Tartaglia Polcini, A., Tripodo, E., Sanchini, P., Gervino, S., Tacconi, D., Dei, S., Mazzierli, M., D’Urso, A., & Ognibene, A. (2020). Comparison of serologic and molecular SARS-CoV 2 results in a large cohort in Southern Tuscany demonstrates a role for serologic testing to increase diagnostic sensitivity. *Clinical Biochemistry*, *84*, 87–92. https://doi.org/10.1016/j.clinbiochem.2020.07.002

65. Paradiso, A. V., De Summa, simona, Silvestris, N., Tommasi, S., Tufaro, A., De Palma, G., Larocca, A. M. V., D’Addabbo, V., Raffaele, D., Cafagna, V., & Garrisi, V. M. (2020). COVID-19 SCREENING AND MONITORING OF ASYMPTOMATIC HEALTH WORKERS WITH A RAPID SEROLOGICAL TEST. *MedRxiv*, 2020.05.05.20086017. https://doi.org/10.1101/2020.05.05.20086017

66. Reiter, T., Pajenda, S., Wagner, L., Gaggl, M., Atamaniuk, J., Holzer, B., Zimpernik, I., Gerges, D., Mayer, K., Aigner, C., Straßl, R., Jansen-Skoupy, S., Födinger, M., Sunder-Plassmann, G., & Schmidt, A. (2020). Covid-19 serology in nephrology health care workers. *MedRxiv*, 2020.07.21.20136218. https://doi.org/10.1101/2020.07.21.20136218

67. Sandri, M. T., Azzolini, E., Torri, V., Carloni, S., Tedeschi, M., Castoldi, M., Mantovani, A., & Rescigno, M. (2020). SARS-CoV-2 serology in 4000 health care and administrative staff across seven sites in Lombardy, Italy. *MedRxiv*. https://doi.org/10.1101/2020.05.24.20111245

68. Santos de Melo, M., Pinto Borges, L., Raguer Valadão de Souza, D., Martins, F., Melquiades de Rezende Neto, J., Alves Ribeiro, A., Santos, A., Bispo da Invenção, G., Leonardo Santos Matos, I., Alves dos, K., Alessandro Alves Souza, N., Chaves Borges, P., Gleydson Brito, M., & Oliveira, de. (2020). Anti-SARS-CoV-2 IgM and IgG antibodies in health workers in Sergipe, Brazil 1 2. *MedRxiv*, 2020.09.24.20200873. https://doi.org/10.1101/2020.09.24.20200873

69. Self, W. H., Tenforde, M. W., Stubblefield, W. B., Feldstein, L. R., Steingrub, J. S., Shapiro, N. I., Ginde, A. A., Prekker, M. E., Brown, S. M., Peltan, I. D., Gong, M. N., Aboodi, M. S., Khan, A., Exline, M. C., Files, D. C., Gibbs, K. W., Lindsell, C. J., Rice, T. W., Jones, I. D., … Zellner, B. (2020). Seroprevalence of SARS-CoV-2 Among Frontline Health Care Personnel in a Multistate Hospital Network — 13 Academic Medical Centers, April–June 2020. *MMWR. Morbidity and Mortality Weekly Report*, *69*(35), 1221–1226. https://doi.org/10.15585/mmwr.mm6935e2

70. Shields, A., Faustini, S. E., Perez-Toledo, M., Jossi, S., Aldera, E., Allen, J. D., Al-Taei, S., Backhouse, C., Bosworth, A., Dunbar, L. A., Ebanks, D., Emmanuel, B., Garvey, M., Gray, J., Kidd, I. M., McGinnell, G., McLoughlin, D. E., Morley, G., O’Neill, J., … Richter, A. G. (2020). SARS-CoV-2 seroprevalence and asymptomatic viral carriage in healthcare workers: A cross-sectional study. *Thorax*, *75*(12), 1089–1094. https://doi.org/10.1136/thoraxjnl-2020-215414

71. Sikora, K., Barwick, I., & Hamilton, C. (2020). Serological prevalence of antibodies to SARS CoV-2 amongst cancer centre staff. *MedRxiv*. https://doi.org/10.1101/2020.05.16.20099408

72. Song, S. K., Lee, D. H., Nam, J. H., Kim, K. T., Do, J. S., Kang, D. W., Kim, S. G., & Cho, M. R. (2020). IgG seroprevalence of COVID-19 among individuals without a history of the coronavirus disease infection in Daegu, Korea. *Journal of Korean Medical Science*, *35*(29). https://doi.org/10.3346/jkms.2020.35.e269

73. Suda, G., Ogawa, K., Kimura, M., Maehara, O., Kitagataya, T., Ohara, M., Tokuchi, Y., Kubo, A., Yamada, R., Shigesawa, T., Suzuki, K., Kawagishi, N., Nakai, M., Sho, T., Natsuizaka, M., Morikawa, K., & Sakamoto, N. (2020). Time-dependent changes in the seroprevalence of COVID-19 in asymptomatic liver disease outpatients in an area in Japan undergoing a second wave of COVID-19. *Hepatology Research*, *50*(10), 1196–1200. https://doi.org/10.1111/hepr.13551

74. Theo Brehm, T., Schwinge, D., Lampalzer, S., Küchen, J., Thompson, M., Ullrich, F., Huber, S., Schmiedel, S., Addo, M. M., Lütgehetmann, M., Schulze zur Wiesch, J., & Lohse, A. W. (2020). High effectiveness of multimodal infection control interventions in preventing SARS-CoV-2 1 infections in healthcare professionals: a prospective longitudinal seroconversion study 2 3. *MedRxiv*, 2020.07.31.20165936. https://doi.org/10.1101/2020.07.31.20165936

75. Torres, J. P., Piñera, C., De La Maza, V., Lagomarcino, A. J., Simian, D., Torres, B., Urquidi, C., Valenzuela, M. T., & O’Ryan, M. (2020). Severe Acute Respiratory Syndrome Coronavirus 2 Antibody Prevalence in Blood in a Large School Community Subject to a Coronavirus Disease 2019 Outbreak: A Cross-sectional Study. *Clinical Infectious Diseases*. https://doi.org/10.1093/cid/ciaa955

76. Torres, P., Empireo, M., Diaque, P., Empireo, G., Rubio, M., Empireo, S., Rodríguez, R., Empireo, S., García, F., Ángel, M., Empireo, L., Saz-Leal, P., & Carlos Del Fresno, I. (2020). *IgG seroprevalence against SARS-CoV-2 in a cohort of 449 non-hospitalized, high-risk exposure individuals*. https://doi.org/10.21203/rs.3.rs-53747/v1

77. Valdivia, A., Torres, I., Huntley, D., Alcaraz, M. J., Albert, E., Solano de la Asunción, C., González, C., Ferrer, J., & Navarro, D. (2020). Caveats in interpreting SARS-CoV-2 IgM+/IgG− antibody profile in asymptomatic health care workers. *Journal of Medical Virology*. https://doi.org/10.1002/jmv.26400

78. Wang, S. mi, Tao, F., Hou, Y., Zhang, A., Xiong, H., Sun, J. jie, Luo, X. ping, Hao, Y., Li, J. xin, Hu, Q., & Liu, A. guo. (2020). Screening of SARS-CoV-2 in 299 Hospitalized Children with Hemato-oncological Diseases: A Multicenter Survey in Hubei, China. *Current Medical Science*, *40*(4), 642–645. https://doi.org/10.1007/s11596-020-2228-7

79. Xiong, S., Guo, C., Dittmer, U., Zheng, X., & Wang, B. (2020). The prevalence of antibodies to SARS-CoV-2 in asymptomatic healthcare workers with intensive exposure to COVID-19. *MedRxiv*. https://doi.org/10.1101/2020.05.28.20110767

80. Xu, R., Huang, J., Duan, C., Liao, Q., Shan, Z., Wang, M., Rong, X., Li, C., Fu, Y., & Wang, H. (2020). Low prevalence of antibodies against SARS-CoV-2 among voluntary blood donors in Guangzhou, China. *Journal of Medical Virology*. https://doi.org/10.1002/jmv.26445

81. Xu, X., Sun, J., Nie, S., Li, H., Kong, Y., Liang, M., Hou, J., Huang, X., Li, D., Ma, T., Peng, J., Gao, S., Shao, Y., Zhu, H., Lau, J. Y. N., Wang, G., Xie, C., Jiang, L., Huang, A., … Hou, F. F. (2020). Seroprevalence of immunoglobulin M and G antibodies against SARS-CoV-2 in China. *Nature Medicine*, *26*(8), 1193–1195. https://doi.org/10.1038/s41591-020-0949-6

82. Zhao, D., Wang, M., Wang, M., Zhao, Y., Zheng, Z., Li, X., Zhang, Y., Wang, T., Zeng, S., Hu, W., Yu, W., & Hu, K. (2020). Asymptomatic infection by SARS-CoV-2 in healthcare workers: A study in a large teaching hospital in Wuhan, China. *International Journal of Infectious Diseases*, *99*, 219–225. https://doi.org/10.1016/j.ijid.2020.07.082
