# Supplementary material for: Seroprevalence of SARS-CoV-2 IgG antibodies among health care workers prior to vaccine administration in Europe, the USA and East Asia: A systematic review and meta-analysis
Source: eClinicalMedicine. 2021 Mar 8;33:100770. doi: 10.1016/j.eclinm.2021.100770 (PMC7938754; doi:10.1016/j.eclinm.2021.100770)
Supplement: Supplementary file 4 [file mmc4.docx]

**Supplementary file 4:** Sensitivity analysis results.

Table of Contents

[Figure A1.1: Overall: Forest plot of the seroprevalence of SARS-CoV-2 antibodies with corresponding 95% confidence intervals (studies with more than 500 HCW 2](#_Toc61984726)

[Figure A1.2: Europe: Forest plot of the seroprevalence of SARS-CoV-2 antibodies with corresponding 95% confidence intervals (studies with more than 500 HCWs). 3](#_Toc61984727)

[Figure A1.3: USA: Forest plot of the seroprevalence of SARS-CoV-2 antibodies with corresponding 95% confidence intervals (studies with more than 500 HCWs). 4](#_Toc61984728)

[Figure A1.4: East Asia: Forest plot of the seroprevalence of SARS-CoV-2 antibodies with corresponding 95% confidence intervals (studies with more than 500 HCWs). 5](#_Toc61984729)

[Figure A1.5: ELISA: Forest plot of the seroprevalence of SARS-CoV-2 antibodies with corresponding 95% confidence intervals (studies with more than 500 HCWs). 6](#_Toc61984730)

[Figure A1.6: Forest plot of the seroprevalence of SARS-CoV-2 antibodies with corresponding 95% confidence intervals (studies with Pre-prints). 7](#_Toc61984731)

# Figure A1.1: Overall: Forest plot of the seroprevalence of SARS-CoV-2 antibodies with corresponding 95% confidence intervals (studies with more than 500 HCW

# Figure A1.2: Europe: Forest plot of the seroprevalence of SARS-CoV-2 antibodies with corresponding 95% confidence intervals (studies with more than 500 HCWs).

# Figure A1.3: USA: Forest plot of the seroprevalence of SARS-CoV-2 antibodies with corresponding 95% confidence intervals (studies with more than 500 HCWs).

# Figure A1.4: East Asia: Forest plot of the seroprevalence of SARS-CoV-2 antibodies with corresponding 95% confidence intervals (studies with more than 500 HCWs).

# Figure A1.5: ELISA: Forest plot of the seroprevalence of SARS-CoV-2 antibodies with corresponding 95% confidence intervals (studies with more than 500 HCWs).

# Figure A1.6: Forest plot of the seroprevalence of SARS-CoV-2 antibodies with corresponding 95% confidence intervals (studies with Pre-prints).
