# Supplementary material for: Seroprevalence of SARS-CoV-2 IgG antibodies among health care workers prior to vaccine administration in Europe, the USA and East Asia: A systematic review and meta-analysis
Source: eClinicalMedicine. 2021 Mar 8;33:100770. doi: 10.1016/j.eclinm.2021.100770 (PMC7938754; doi:10.1016/j.eclinm.2021.100770)
Supplement: Supplementary file 5 [file mmc5.docx]

**Supplementary file 5:** Factors associated with SARS-CoV-2 IgG antibodies positivity.

## Forest plot exhibiting Odds Ratio (OR) of each study and combined OR of the crude association between gender and IgG positivity.

## Forest plot exhibiting Odds Ratio (OR) of each study and combined OR of the crude association between age-group and IgG positivity.

## Forest plot exhibiting Odds Ratio (OR) of each study and combined OR of the crude association between level of high-risks HCWs and IgG positivity.
