# Supplementary material for: Seroprevalence of SARS-CoV-2 IgG antibodies among health care workers prior to vaccine administration in Europe, the USA and East Asia: A systematic review and meta-analysis
Source: eClinicalMedicine. 2021 Mar 8;33:100770. doi: 10.1016/j.eclinm.2021.100770 (PMC7938754; doi:10.1016/j.eclinm.2021.100770)
Supplement: Supplementary file 6 [file mmc6.docx]

**Supplementary file 6:** Funnel plots are widely used in meta-analysis to visually assess small study effects. We found five of studies had publication bias (9.6%), which indicates most points fall within the 95% confidence region. Each point represents a study. One plot shows the real-data funnel plot at a randomly chosen position and 3 plots show data simulated under some suitable null hypothesis. Egger’s regression line is drawn in each funnel plot and imputed studies potentially missing due to publication bias are shown by black symbols.
